# Human‐to‐Human Transmission of Severe Fever With Thrombocytopenia Syndrome Resulting in Fatal Cases: A Case Series

**DOI:** 10.1155/crdi/5597862

**Published:** 2026-02-02

**Authors:** Lu Yao, Xiaobo Yang, Xuehui Gao, Yin Yuan, Chang Li, Chenggang Gao, Huaqing Shu, Xiaojing Zou, Ruiting Li, Jiqian Xu, You Shang

**Affiliations:** ^1^ Department of Critical Care Medicine, Union Hospital, Tongji Medical College, Huazhong University of Science and Technology, Wuhan, 430022, China, hust.edu.cn; ^2^ Department of Critical Care Medicine, West China Hospital, Sichuan University, Chengdu, 610041, China, scu.edu.cn

**Keywords:** clinical management, diagnostic delay, elderly, human-to-human transmission, SFTS

## Abstract

Severe fever with thrombocytopenia syndrome (SFTS), primarily a tick‐borne disease, can also cause fatal human‐to‐human transmission. This report analyzes a cluster of six SFTS cases identified in China in 2022, involving one index patient and five secondary infections, with an overall mortality of 83%. All secondary cases occurred in elderly individuals (aged 66–85 years) following unprotected exposure to the index patient’s body fluids during bedside care or traditional postmortem rituals, without documented tick bites. The high fatality rate underscores the potential severity of secondary transmission, particularly among elderly adults. More critically, this outbreak exposes systemic delays in early diagnosis even within an endemic area, highlighting fundamental gaps in the clinical management of undifferentiated fever. Effective prevention, therefore, relies on establishing a clinical system for early detection, rapid diagnosis, and prompt isolation while implementing culturally adapted community interventions to reliably interrupt transmission.

## 1. Introduction

Dabie bandavirus (DBV), a novel phlebovirus, is the etiological agent responsible for severe fever with thrombocytopenia syndrome (SFTS). The virus circulates among several host species, including patients, livestock, and poultry [1]. While the primary transmission route of SFTS is through tick bites [2], emerging evidence indicates that direct zoonotic transmission, with notable risks posed by contact with ill or deceased cats and dogs, is a growing public health concern in endemic areas [3, 4]. Furthermore, human‐to‐human transmission has been documented through exposure to patients’ blood or bodily fluids [5, 6]. The detection of DBV RNA in semen also suggests a potential risk of sexual transmission [7], and aerosol transmission has been proposed as an additional possible route [8]. Although SFTS cases typically present sporadically, rare cluster outbreaks have been reported [9]. Individuals residing in or visiting endemic regions, especially those with close contact with patients or infected animals, constitute the primary population at risk [2].

Clinically, SFTS is characterized by fever, gastrointestinal symptoms, thrombocytopenia, and leukopenia. The disease generally progresses through three phases: febrile, multiorgan dysfunction, and convalescence. Although most patients experience a self‐limiting course with gradual normalization of laboratory parameters, a subset rapidly progresses to multiple organ failure and death [10]. The high case‐fatality rate and the absence of specific antiviral treatments have made SFTS an emerging global health threat [11].

From April to June 2022, a cluster of six confirmed SFTS cases resulting from human‐to‐human transmission was identified in an endemic area of China. The index patient, a 73‐year‐old male with fever, nausea, leukopenia, and thrombocytopenia, succumbed to the disease three days after hospitalization. Following exposure to the index patient’s blood or bodily fluids, both before and after his death, five secondary cases developed fever and other symptoms, four of whom subsequently died. Here, we report the epidemiological and clinical findings from this fatal cluster, with a particular focus on analyzing the systemic vulnerabilities in early case identification that contributed to its occurrence and severity.

## 2. Cases Presentation

On April 29, 2022, the index patient, a 73‐year‐old male, developed a fever (maximum body temperature, 39.0°C) without any specific incentives, accompanied by chills, fatigue, anorexia, and nausea. He sought treatment at a local hospital on May 2, 2022. A routine blood test revealed leukopenia (white blood cell count, 2.0 × 10^9^/L) and thrombocytopenia (platelet count, 50 × 10^9^/L). He received no treatment at the local hospital and was admitted to the central hospital on the same day. Examination upon admission confirmed leukopenia, thrombocytopenia, coagulation dysfunction, and hepatic insufficiency. A laboratory test for DBV was positive, confirming the diagnosis of SFTS. Despite clinical management, his condition showed no improvement. On the morning of May 4, 2022, the patient became confused, unresponsive, and suffered a grand mal seizure. Later that evening, he experienced tachypnea, decreased blood oxygen saturation, and hypotension. Airway intubation with mechanical ventilation was initiated to improve oxygenation, and vasoactive drugs were administered to stabilize blood pressure. During this period, he received adjunctive treatments including antibiotics, antivirals, corticosteroids, and bedside blood purification. Nonetheless, his condition continued to deteriorate, manifested by massive airway bleeding, a progressive decline in red blood cells and hemoglobin, and an inability to maintain circulation. After being informed of the situation, the family requested the withdrawal of life‐sustaining treatment. The index patient was discharged from the hospital on May 5, 2022, and passed away within a few hours of returning home.

The five secondary cases comprised one hospital visitor and four family members involved in postmortem rituals. Case 1, a 68‐year‐old male, visited the index patient in the hospital ward on May 4, 2022, and provided close physical care that included repeatedly wiping the patient’s face and oral secretions with his bare hands, without any personal protective equipment. Following the patient’s death, the four other secondary cases participated in the traditional washing and preparation of the body in accordance with local customs. During this unprotected process, Case 2 (index patient’s elder brother, an 85‐year‐old male) and Case 3 (index patient’s distant nephew, a 66‐year‐old male) were primarily responsible for cleansing the body, resulting in direct skin‐to‐skin contact with blood and other body fluids. Concurrently, Case 4 (index patient’s in‐law, a 72‐year‐old male) and Case 5 (index patient’s uncle, an 80‐year‐old male) assisted by handling and changing the soiled bedding and clothing, which were heavily contaminated with blood and secretions.

**Figure 1:**
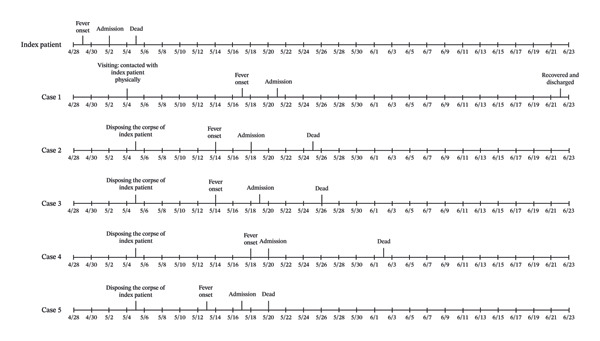
Timeline of key events for SFTS patients.

All five secondary cases had no history of definite tick bites. They developed symptoms within 8 to 13 days following exposure to the index patient. Tragically, only Case 1 survived; the remaining four patients died within 15 days of symptom onset. Laboratory testing confirmed the presence of DBV RNA in all five secondary cases, confirming the diagnosis of SFTS. All secondary cases were of advanced age, and apart from Case 1, all had various underlying diseases. Fever was the initial symptom in all five, with fatigue, myalgia, nausea, and diarrhea being common upon admission. Case 1 was admitted with hemorrhagic and central nervous system symptoms but had a more favorable prognosis. All five cases presented with leukopenia and thrombocytopenia upon admission and developed multiorgan dysfunction during their clinical course. Table 1 outlines detailed clinical information for all patients, and Figure 1 depicts a timeline of critical events.

**Table 1:** Clinical characteristics of SFTS patients.

	Index patient	Case 1	Case 2	Case 3	Case 4	Case 5
*General information*						
Age, years	68	68	85	66	72	80
Gender	Male	Male	Male	Male	Male	Male
Relationship with index patient	NA	Brother‐in‐law	Elder brother	Distant nephew	Relatives by marriage	Uncle
Underlying diseases	Healthy	Healthy	Pulmonary tuberculosis	COPD	Chronic enteritis	Hypertension

*Key events and outcomes*						
Date of onset	April 29, 2022	May 17, 2022	May 14, 2022	May 14, 2022	May 18, 2022	May 13, 2022
Date of admission	May 2, 2022	May 21, 2022	May 18, 2022	May 19, 2022	May 20, 2022	May 17, 2022
Days from onset to admission	3	4	4	5	2	4
Date of sample collection	May 3, 2022	May 26, 2022	May 24, 2022	May 26, 2022	May 22, 2022	May 19, 2022
Days from onset to sampling	4	9	10	12	4	6
Clinical outcome	Dead	Recovered	Dead	Dead	Dead	Dead
Date of death	May 5, 2022	NA	May 25, 2022	May 26, 2022	June 2, 2022	May 20, 2022
Days of hospitalization	3	32	7	7	13	3
Days from onset to death	6	NA	11	12	15	7

*Clinical symptoms and signs on admission*						
Fever	Yes	Yes	Yes	Yes	Yes	Yes
Rigor	Yes	No	No	No	No	Yes
Fatigue	Yes	Yes	Yes	No	Yes	Yes
Myalgia	No	Yes	No	Yes	Yes	Yes
Lymphadenopathy	No	No	No	No	Yes	No
Anorexia	Yes	No	Yes	No	Yes	Yes
Nausea	Yes	Yes	Yes	No	Yes	Yes
Vomiting	No	No	Yes	No	Yes	No
Diarrhea	No	Yes	No	Yes	Yes	No
Cough and expectoration	No	No	No	No	No	Yes
Oral hemorrhage	No	Yes	No	No	No	No
Dizziness	No	No	Yes	No	No	Yes
Headache	No	No	Yes	No	No	Yes
Lethargy	No	Yes	No	No	No	No

*Laboratory findings on admission*						
PLT (× 10^9^/L)	45	38	108	22	81	89
WBC (× 10^9^/L)	1.38	3.75	2.1	1.1	1.8	2.8
APTT (s)	69.2	63.6	NA	62.9	34.1	38
PT (s)	14.55	14.3	NA	13.6	13.5	15.6
FIB (g/L)	1.79	1.41	NA	1.86	2.07	2.53
ALT (U/L)	177	96	21	63	20	18
AST (U/L)	599	399	51	166	24	24
LDH (U/L)	1163	1372	324	679	100	193
CK (U/L)	3887	2025	117	744	147	73
Cr (μmol/L)	97	67.6	89	88.3	80	78
BUN (mmol/L)	7.54	4.93	9.41	4.78	6.17	5.67
MODS	Yes	Yes	Yes	Yes	Yes	Yes

*Virological testing*						
Real‐time RT‐PCR (Ct value)	25.47	No data	24.63	No data	28.58	26.68
DBV load (TCID_50_/mL)	No data	9.22 × 10^3^	No data	1.13 × 10^3^	No data	No data

*Note:* PLT, platelet; FIB, fibrinogen; ALT, alanine transaminase; AST, aspartate aminotransferase; LDH, lactic dehydrogenase; Cr, creatinine; DBV, Dabie bandavirus.

Abbreviations: APTT, activated partial thromboplastin time; BUN, blood urea nitrogen; CK, creatine kinase; COPD, chronic obstructive pulmonary disease; Ct, cycle threshold; MODS, multiple organ dysfunction syndrome; NA, not available; PT, prothrombin time; RT‐PCR, reverse transcription polymerase chain reaction; TCID_50_, 50% tissue culture infectious dose; WBC, white blood cell.

No further transmission was documented during the two‐month follow‐up. Specifically, the index patient’s son (39 years old) and grandson (16 years old) remained asymptomatic despite close contact during hospitalization. All close contacts of the secondary patients also showed no SFTS‐related symptoms; blood samples obtained from three consenting individuals were negative for DBV RNA. In addition, no infections occurred among the attending physicians or nurses.

## 3. Discussion

A cluster of severe and fatal SFTS cases resulting from human‐to‐human transmission is detailed here, revealing a significant and often overlooked public health risk. While SFTS is primarily a tick‐borne disease, and recent evidence highlights the role of infected companion animals like cats and dogs in its transmission, our findings emphasize that direct contact with SFTS patients or their remains constitutes a potent mechanism for outbreak propagation.

This cluster of cases highlights the risk of human‐to‐human SFTS transmission while simultaneously exposing systemic delays in early detection and diagnosis within the local healthcare framework. Despite the region being endemic for SFTS, a considerable lag occurred between symptom onset and confirmed diagnosis in the index patient, pointing to critical weaknesses in the existing diagnostic and clinical management framework for undifferentiated fever cases where SFTS should be suspected. Multiple factors constrain timely diagnosis in these settings, including limited access to rapid pathogen testing at the primary care level, inadequate prioritization of SFTS in clinical differential diagnosis, and systemic inefficiencies in identifying, reporting, and referring cases. Such framework‐level deficiencies directly result in missed opportunities for early isolation and intervention, facilitating further viral spread through family caregiving and high‐risk funeral practices. Consequently, a central implication of this outbreak is that the most pressing need in affected areas is to develop and implement a robust clinical framework to enhance early suspicion, rapid diagnosis, and standardized management of potential SFTS cases, a fundamental step toward preventing similar transmission chains.

In the present cluster, the transmission chain was unequivocally linked to unprotected exposure to the index patient’s body fluids. Case 1 acquired the infection through direct, unprotected contact with oral secretions during bedside care. Crucially, the other four secondary cases were infected solely through their involvement in postmortem rituals, handling the body, and contaminated fomites without any personal protective equipment. This aligns with evidence indicating that SFTS patients remain infectious via blood and secretions for a substantial period after symptom onset [12] and that corpses can harbor the virus, posing a prolonged transmission risk [13]. Although environmental or aerosol transmission has been proposed [14], the absence of infections among healthcare workers and other family members who had no direct fluid exposure strongly supports direct contact as the dominant route in this cluster.

A notable aspect of this outbreak is the marked disparity in infection outcomes among exposed individuals. While the index patient’s son and grandson participated in caregiving during hospitalization, they likely avoided infection due to the absence of high‐risk fluid exposure. In contrast, all other affected relatives were infected during postmortem rituals through unprotected contact with the body or contaminated materials. Such variation can be attributed not only to differing exposure intensity but also to a clear risk gradient across the contact settings involved. In the hospital environment, even nonprofessional caregivers may benefit from contextual constraints and nascent protective awareness, thereby reducing exposure risk. Conversely, in community funeral settings entirely removed from medical safeguards, traditional practices often override infection control, creating high‐risk nodes for transmission. These high‐risk scenarios underscore a critical transition point in which patients or human remains move from controlled healthcare settings back into the community, and the attending physician bears decisive responsibility. Failure to ensure adequate community preparedness or to provide clear infection control guidance to medically untrained families constitutes a professional breach that directly facilitates viral spread into the community and complicates containment efforts. As this infection cluster demonstrates, preventing secondary transmission relies fundamentally on physicians fulfilling their educational duty prior to discharge. Neglecting systematic prerelease instruction and coordination represents not merely an oversight but an abandonment of their core duty to protect community health, a failure that can amplify isolated cases into localized outbreaks. Thus, physician‐led education at the transition point remains essential to both clinical ethics and effective public health practice, necessitating a deliberate extension of professional responsibility beyond institutional boundaries toward active community safeguarding.

In contrast, all five older secondary patients, who sustained intensive fluid contact, developed the disease. This discrepancy underscores the critical role of exposure intensity and duration in determining transmission risk [15]. Furthermore, it highlights the influence of host factors. The four secondary patients who died were all elderly (aged 66–85 years) with significant comorbidities, whereas the sole survivor (Case 1) was relatively younger and healthier. Advanced age is a well‐established predictor of poor outcomes in SFTS, likely due to immunosenescence and a diminished capacity to mount an effective antiviral response [16, 17]. This may explain the divergence between our observations, in which comorbidities were common in fatal cases, and some previous reports [18], suggesting that underlying conditions may exacerbate the vulnerability conferred by advanced age. Notably, the clinical course in these secondary cases was remarkably severe, contradicting earlier reports that suggested secondary transmissions often lead to milder disease [19–21]. The rapid progression to multiorgan dysfunction and the high case‐fatality rate observed here serve as a critical reminder that human‐to‐human transmission can result in devastating outcomes, particularly in older populations with compromised health.

## 4. Conclusion

By revealing both the severe consequences of human‐to‐human SFTS transmission and the systemic weaknesses in early diagnosis within endemic regions, this fatal cluster underscores the urgent need for a strengthened clinical framework. Effective prevention requires building a system that prioritizes early diagnostic capacity through enhanced primary care vigilance, optimized rapid testing and differential diagnosis for SFTS, and prompt case detection and isolation. Integrating these measures with strict healthcare infection control and culturally adapted community interventions can effectively disrupt transmission chains and prevent future outbreaks.

## Author Contributions

Lu Yao conceived the study, collected clinical data, and drafted the manuscript. Xiaobo Yang conducted literature review and assisted in manuscript preparation and critical revision. Xuehui Gao and Yin Yuan performed data validation, with Xuehui Gao additionally contributing to formal analysis. Chang Li and Chenggang Gao were responsible for longitudinal case follow‐up and resource management. Huaqing Shu, Xiaojing Zou, and Ruiting Li provided clinical resources and expertise for diagnosis and treatment. Jiqian Xu and You Shang supervised the research framework and critically revised the manuscript for intellectual content.

## Funding

The authors have no funding to disclose.

## Disclosure

All authors agree to be accountable for the research presented.

## Ethics Statement

Ethics approval was waived for this retrospective case series as it describes standard clinical care using fully deidentified patient data. All identifying information was removed to protect patient confidentiality.

## Conflicts of Interest

The authors declare no conflicts of interest.

## Data Availability

All data generated during this study are included in this published article.

## References

[1] Yu X. J. , Liang M. F. , Zhang S. Y. et al., Fever with Thrombocytopenia Associated with a Novel Bunyavirus in China, New England Journal of Medicine. (2011) 364, no. 16, 1523–1532, 10.1056/nejmoa1010095, 2-s2.0-79955166070. 21410387 PMC3113718

[2] Liu Q. , He B. , Huang S. Y. , Wei F. , and Zhu X. Q. , Severe Fever with Thrombocytopenia Syndrome, an Emerging tick-borne Zoonosis, The Lancet Infectious Diseases. (2014) 14, no. 8, 763–772, 10.1016/s1473-3099(14)70718-2, 2-s2.0-84904720901. 24837566

[3] Kobayashi Y. , Kato H. , Yamagishi T. et al., Severe Fever with Thrombocytopenia Syndrome, Japan, 2013–2017, Emerging Infectious Diseases. (2020) 26, no. 4, 692–699, 10.3201/eid2604.191011. 32186502 PMC7101122

[4] Yamanaka A. , Kirino Y. , Fujimoto S. et al., Direct Transmission of Severe Fever with Thrombocytopenia Syndrome Virus from Domestic Cat to Veterinary Personnel, Emerging Infectious Diseases. (2020) 26, no. 12, 2994–2998, 10.3201/eid2612.191513. 33219655 PMC7706950

[5] Jung I. Y. , Choi W. , Kim J. et al., Nosocomial person-to-person Transmission of Severe Fever with Thrombocytopenia Syndrome, Clinical Microbiology and Infections. (2019) 25, no. 5, 10.1016/j.cmi.2019.01.006, 2-s2.0-85061607520. 30677496

[6] Gong L. , Song D. D. , Wu J. B. et al., Human-To-Human Transmissions of Severe Fever with Thrombocytopenia Syndrome Virus in Anhui Province, 2010-2017, Clinical Microbiology and Infections. (2018) 24, no. 8, 920–922, 10.1016/j.cmi.2018.03.014, 2-s2.0-85050118671. 29559391

[7] Koga S. , Takazono T. , Ando T. et al., Severe Fever with Thrombocytopenia Syndrome Virus RNA in Semen, Japan, Emerging Infectious Diseases. (2019) 25, no. 11, 2127–2128, 10.3201/eid2511.190061, 2-s2.0-85073616498. 31625854 PMC6810197

[8] Gong Z. , Gu S. , Zhang Y. et al., Probable Aerosol Transmission of Severe Fever with Thrombocytopenia Syndrome Virus in Southeastern China, Clinical Microbiology and Infections. (2015) 21, no. 12, 1115–1120, 10.1016/j.cmi.2015.07.024, 2-s2.0-84941754763. 26255811

[9] Chen H. , Hu K. , Zou J. , and Xiao J. , A Cluster of Cases of human-to-human Transmission Caused by Severe Fever with Thrombocytopenia Syndrome Bunyavirus, International Journal of Infectious Diseases. (2013) 17, no. 3, e206–e208, 10.1016/j.ijid.2012.11.006, 2-s2.0-84874465346. 23218674

[10] Gai Z. T. , Zhang Y. , Liang M. F. et al., Clinical Progress and Risk Factors for Death in Severe Fever with Thrombocytopenia Syndrome Patients, The Journal of Infectious Diseases. (2012) 206, no. 7, 1095–1102, 10.1093/infdis/jis472, 2-s2.0-84866132492. 22850122

[11] Liang S. Y. , Chu H. L. , Guo X. L. et al., Experimental Infections of Mosquitoes with Severe Fever with Thrombocytopenia Syndrome Virus, Infectious Diseases of Poverty. (2017) 6, no. 1, 10.1186/s40249-017-0282-6, 2-s2.0-85020031228. PMC5452404 28569189

[12] Fang X. , Hu J. , Peng Z. et al., Epidemiological and Clinical Characteristics of Severe Fever with Thrombocytopenia Syndrome Bunyavirus human-to-human Transmission, PLoS Tropical Disease. (2021) 15, no. 4, 10.1371/journal.pntd.0009037. PMC8087050 33930022

[13] Gai Z. , Liang M. , Zhang Y. et al., Person-To-Person Transmission of Severe Fever with Thrombocytopenia Syndrome Bunyavirus Through Blood Contact, Clinical Infectious Diseases. (2011) 54, no. 2, 249–252, 10.1093/cid/cir776, 2-s2.0-84555204783. 22095565 PMC3245727

[14] Ryu B. H. , Kim J. Y. , Kim T. et al., Extensive Severe Fever with Thrombocytopenia Syndrome Virus Contamination in Surrounding Environment in Patient Rooms, Clinical Microbiology and Infections. (2018) 24, no. 8, 911.e1–911.e4, 10.1016/j.cmi.2018.01.005, 2-s2.0-85042167398. 29355730

[15] Bao C. J. , Guo X. L. , Qi X. et al., A Family Cluster of Infections by a Newly Recognized Bunyavirus in Eastern China, 2007: Further Evidence of person-to-person Transmission, Clinical Infectious Diseases. (2011) 53, no. 12, 1208–1214, 10.1093/cid/cir732, 2-s2.0-81855166035. 22028437

[16] Gavazzi G. and Krause K. H. , Ageing and Infection, The Lancet Infectious Diseases. (2002) 2, no. 11, 659–666, 10.1016/s1473-3099(02)00437-1, 2-s2.0-0036840992. 12409046

[17] Seo J. W. , Kim D. , Yun N. , and Kim D. M. , Clinical Update of Severe Fever with Thrombocytopenia Syndrome, Viruses. (2021) 13, no. 7, 10.3390/v13071213. PMC8310018 34201811

[18] Wang Y. , Song Z. , Xu X. et al., Clinical Symptoms Associated with Fatality of Severe Fever with Thrombocytopenia Syndrome: a Systematic Review and meta-analysis, Acta Tropical. (2022) 232, 10.1016/j.actatropica.2022.106481. 35461803

[19] Jia B. , Wu W. , Huang R. et al., Characterization of Clinical Features and Outcome for human-to-human Transmitted Severe Fever with Thrombocytopenia Syndrome, Infectious Disease (Lond).(2018) 50, no. 8, 601–608, 10.1080/23744235.2018.1449962, 2-s2.0-85044076932. 29542384

[20] Wang Y. , Deng B. , Zhang J. , Cui W. , Yao W. , and Liu P. , Person-To-Person Asymptomatic Infection of Severe Fever with Thrombocytopenia Syndrome Virus Through Blood Contact, Internal Medicine. (2014) 53, no. 8, 903–906, 10.2169/internalmedicine.53.1164, 2-s2.0-84898639275. 24739616

[21] Wu Y. X. , Yang X. , Leng Y. et al., Human-To-Human Transmission of Severe Fever with Thrombocytopenia Syndrome Virus Through Potential Ocular Exposure to Infectious Blood, International Journal of Infectious Diseases. (2022) 123, 80–83, 10.1016/j.ijid.2022.08.008. 35987469

